# Draft Genome Sequences of 16 Strains of Escherichia Cryptic Clade II Isolated from Intertidal Sediment in Hong Kong

**DOI:** 10.1128/MRA.00416-19

**Published:** 2019-07-18

**Authors:** Zhiyong Shen, Xiu Pei Koh, Yanping Yu, Chun Fai Woo, Yigang Tong, Stanley C. K. Lau

**Affiliations:** a Department of Ocean Science, The Hong Kong University of Science and Technology, Clear Water Bay, Hong Kong; b Division of Environment and Sustainability, The Hong Kong University of Science and Technology, Clear Water Bay, Hong Kong; c State Key Laboratory of Pathogen and Biosecurity, Beijing, China; University of Maryland School of Medicine

## Abstract

The genus Escherichia includes several cryptic clades. Among them, the members of cryptic clade II have rarely been found, and their genome sequences remain largely uninvestigated. Here, we report the draft genome sequences of 16 strains of *Escherichia* cryptic clade II that were isolated from intertidal sediment in Hong Kong.

## ANNOUNCEMENT

Escherichia is currently comprised of four validly published species (E. albertii, E. coli, E. fergusonii, and E. marmotae) and a number of genetically divergent yet taxonomically inconspicuous monophyletic lineages, commonly referred to as cryptic clades. The distribution, prevalence, ecological niches, and genomic features of the cryptic clades have been investigated in a number of studies (1–3). However, due to the scarcity of its isolates, the genomic composition of cryptic clade II remains largely uninvestigated, leaving a knowledge gap about the evolutionary history, ecological character, and evolution of the *Escherichia* genus as a whole. Here, we report the draft genome sequences of 16 strains of cryptic clade II, isolated from the intertidal sediment in the subtropical environment of Hong Kong.

The strains were isolated using the selective medium CHROMagar ECC (CHROMagar, France) and putatively identified as E. coli on the basis of their growth on the medium as blue colonies. However, in a maximum likelihood phylogenetic tree constructed using the concatenated DNA sequences of seven housekeeping genes, *adk*, *fumC*, *gyrB*, *icd*, *mdh*, *purA*, and *recA* (4), the 16 strains occupied the same monophyletic lineage as that occupied by previously reported members of cryptic clade II (1) instead of being affiliated with E. coli.

To obtain the genome sequences of the 16 strains of cryptic clade II, genomic DNA extracted from overnight cultures in Luria-Bertani broth was sheared using Ion Shear Plus reagents, end repaired, and ligated to Ion Torrent adapters (Life Technologies, USA). Libraries containing fragments ca. 400 bp in length were sequenced on an Ion Torrent platform to generate single-ended reads. The sequence reads were processed and analyzed using software with default parameters, as described below.

**FIG 1:**
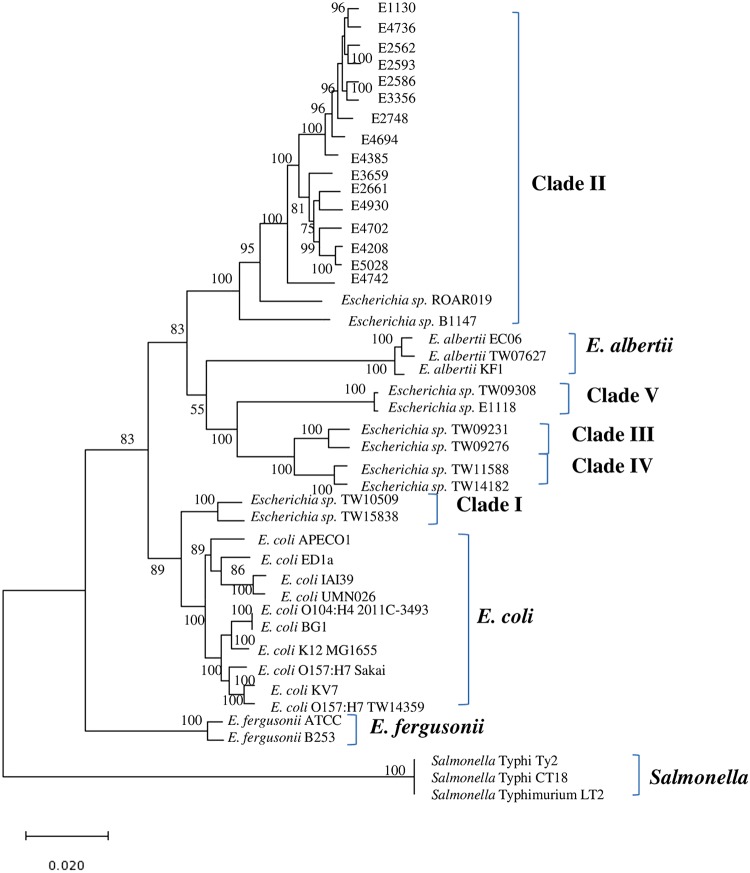
A maximum likelihood phylogenetic tree of 405 core genes extracted from 44 strains of *Escherichia* and *Salmonella*. The concentenated sequences of the core genes were aligned using MUSCLE (11). The tree was constructed by using MEGA7 (12) with the Jukes-Cantor subsitution model, the nearest-neighbor interchange topology search strategy, and 100-bootstrap replication. Numbers at the nodes indicate bootstrap values that are greater than 50. The scale bar indicates substitutions per nucleotide position.

Briefly, raw reads were filtered for quality using FastQC (Q > 30). Clean reads without adapter sequences were *de novo* assembled into contigs using MIRA v4.0.2 (5) and SPAdes v3.6.0 (6) on the SIMBA Workbench platform (7). Assembly quality was enhanced through (i) the mapping of contigs to reference genomes by using CONTIGuator (8) and the optical mapping reports generated by MapSolver (OpGen, Inc.), (ii) the determination of the origins in circular genomes by using the moveDNAA.py script, and (iii) the manual closure of gaps through the identification of repeats on the extremities of the contig using BLAST (9). After the removal of small contigs (<500 bp), the genome sequences obtained for the 16 strains were 4,687,583 to 5,244,655 bp (Table 1). *N*_50_ values were all greater than 100 kb. Except for strain E4742, the genome assembly of each strain contains less than 100 contigs. The genome sequences were annotated using the NCBI Prokaryotic Genome Annotation Pipeline. In a maximum likelihood phylogenetic tree constructed for the core genes in *Escherichia* spp. (Fig. 1), the 16 strains and the previously reported members of cryptic clade II (i.e., *Escherichia* sp. strains ROAR019 [10] and B1147 [1]) occupied a monophyletic lineage, congruent to the phylogeny inferred on the basis of seven housekeeping genes.

**TABLE 1:** Summary of draft genome assemblies

Strain	Total no. of reads	Read length (bp)	Coverage (×)	Assembly size (bp)	No. of contigs	*N*_50_ (bp)	G+C content (%)	No. of CDS*^a^*	No. of tRNA coding genes	GenBank accession no.	Sequence Read Archive accession no.
E1130	1,176,251	35–400	50	5,244,655	62	383,389	50.71	5,040	77	PDIL00000000	SRX3260321
E2562	1,280,272	35–400	50	4,940,096	49	332,841	50.47	4,724	85	PDIJ00000000	SRX3260343
E2586	1,102,521	35–400	45	5,094,594	78	364,248	50.47	5,232	77	PDIH00000000	SRX3260344
E2593	1,428,384	35–400	60	5,176,532	78	173,826	50.41	5,417	72	PDII00000000	SRX3260345
E2661	1,047,543	35–400	40	4,955,692	95	183,982	50.73	4,952	79	PDIG00000000	SRX3260346
E2748	1,158,520	35–400	45	4,837,679	60	314,245	50.61	4,788	76	PDIF00000000	SRX3260347
E3356	1,104,182	35–400	40	4,918,899	49	389,954	50.55	4,800	76	PDIE00000000	SRX3260348
E3659	973,907	35–400	40	5,033,702	76	136,855	50.60	4,886	73	PDID00000000	SRX3260350
E4208	1,041,529	35–400	45	4,687,583	57	135,714	50.81	4,762	81	PDIC00000000	SRX3260359
E4385	1,796,955	35–400	80	5,015,426	43	190,271	50.59	5,105	75	PDIK00000000	SRX5623432
E4694	1,179,794	35–400	50	4,891,728	36	361,728	50.62	4,575	76	PDIB00000000	SRX3260358
E4702	1,145,459	35–400	50	5,114,924	73	219,627	50.75	4,764	80	PDIA00000000	SRX3260360
E4736	1,879,325	35–400	100	4,844,105	43	310,513	50.50	4,453	67	PDHX00000000	SRX3200104
E4742	1,270,039	35–400	50	5,195,263	113	177,505	50.60	4,897	89	PDHY00000000	SRX3260361
E4930	1,886,454	35–400	100	5,119,612	78	247,834	50.60	4,767	79	PDHZ00000000	SRX3260362
E5028	1,787,838	35–400	100	5,050,713	91	147,572	50.50	4,596	84	PDHW00000000	SRX3268009

a CDS, coding DNA sequences.

### Data availability.

The genome assemblies have been deposited in DDBJ/ENA/GenBank under BioProject number PRJNA412557 with accession numbers from PDHW00000000 to PDIL00000000 (Table 1).
